# Current Approaches of Nuclear Molecular Imaging in Breast Cancer

**DOI:** 10.3390/cancers17132105

**Published:** 2025-06-23

**Authors:** Laura Schäfer, Betül Altunay, Amelie Heesch, Thiemo van Nijnatten, Sofia Vaz, Malik Eid Juweid, Felix Manuel Mottaghy

**Affiliations:** 1 Department of Nuclear Medicine, University Hospital RWTH Aachen, 52074 Aachen, Germany; laschaefer@ukaachen.de (L.S.); baltunay@ukaachen.de (B.A.); aheesch@ukaachen.de (A.H.); 2 Department of Radiology and Nuclear Medicine, Maastricht University Medical Center (MUMC+), 6229 HX Maastricht, The Netherlands; thiemo.nijnatten@mumc.nl; 3 Nuclear Medicine-Radiopharmacology, Champalimaud Clinical Center, Champalimaud Foundation, 1400-038 Lisbon, Portugal; sofia.vaz@fundacaochampalimaud.pt; 4 Leiden University Medical Center, 2333 ZA Leiden, The Netherlands; 5 Department of Radiology and Nuclear Medicine, University of Jordan, Amman 11942, Jordan; mjuweid@yahoo.com

**Keywords:** breast cancer, PET, molecular imaging, prediction, precision medicine

## Abstract

In this narrative review, we aim to summarize recent advancements in nuclear molecular imaging techniques and their applications in BCa management. The main focus of this review is on the most relevant clinical investigations from the past 2–3 years that highlight the enhanced diagnostic and therapeutic value of these imaging modalities. A variety of radiolabeled probes introduced in molecular imaging is explored, detailing their roles in the accurate diagnosis and characterization of BCa, as well as their integration into radioligand therapy and theragnostic strategies.

## Simple Summary

Breast cancer (BCa) is the most prevalent malignancy among women worldwide, representing a significant public health challenge due to its complex biology, varied subtypes, and the need for tailored therapeutic strategies. Early and accurate diagnosis is critical for improving patient outcomes. Molecular imaging has emerged as a transformative approach in oncology, enabling the visualization of biological processes at the molecular and cellular levels. This technique utilizes radiolabeled probes to target specific biomarkers associated with cancer, facilitating the detection of tumors and metastases with greater precision.

## 1. Introduction

Breast cancer (BCa) is globally the most prevalent cancer in women. Additionally, there are 43,780 BCa associated deaths annually, positioning it as the fifth highest cause of mortality on a global scale [1]. Notably, BCa mortality rates are higher in transitioning countries, such as those in Melanesia and Western Africa, compared to transitioned countries like Australia, Western Europe, and Northern America [2]. Moreover, female sex, older age, family history, and genetic mutations are significant risk factors [2,3,4]. In a general analysis, 87% of patients are still alive 5 years after diagnosis [5]. However, in BCa, there may still be a risk of local recurrences or metastases even after a longer period, necessitating long-term follow-ups unlike many other cancer types [5]. Treatment options and prognosis vary depending on the cancer subtype and stage. The classification of different subtypes is primarily based on receptor expression status, which serves as a target for personalized treatment approaches [6]. The main receptors in this context are the estrogen receptor (ER), the progesterone receptor (PR), and the human epidermal growth factor receptor 2 (HER2).

Approximately 15% of BCa cases do not express ER, PR, or HER2, categorizing them as triple-negative BCa (TNBC) [7]. This highly aggressive subtype is associated with an earlier age of onset compared to other BCa subtypes. Additionally, TNBC tumors are typically larger and higher grade at the time of diagnosis than other subtypes. They often present with poorly differentiated histology and have a propensity for lymph node metastases, with a higher likelihood of metastasizing to the lung and brain [8,9]. The absence of the three receptors poses a challenge in directly addressing TNBC cells. The diversity of TNBC is demonstrated through its six molecular subtypes, comprising two basal-like, an immunomodulatory, a mesenchymal, a mesenchymal stem-like, and a luminal androgen receptor subtype. Each subtype presents distinct characteristics such as modified biological pathways, genetic mutations, and varying cell compositions [10]. Clinical interventions for TNBC involve cytotoxic chemotherapy, radiation therapy, and surgical procedures [7]. However, due to the non-specific nature of these treatments, their effectiveness is limited, still resulting in a less favorable prognosis for TNBC patients [11,12]. To enhance the long-term survival for patients, it is imperative to conduct precise molecular and cellular analyses to comprehend the origin and progression of this disease.

The field of nuclear medicine, encompassing various disciplines, is increasingly pivotal in oncology. Radiolabeled chemical compounds, also called radiotracers or more precisely radiopharmaceuticals, now play a crucial role in non-invasive cancer diagnosis, staging, therapy monitoring, and treatment. These radiotracers comprise various radionuclides linked to molecules that target specific tumor-associated receptors, transmembraneous channels, pathways or enzymes, as well as features of the tumor microenvironment (TME). The breast TME consists of a complex network of extracellular matrix and diverse cell types, including epithelial cells, endothelial cells, immune cells, fibroblasts, and adipocytes [13]. During tumor progression, changes such as enhanced angiogenesis, activation of fibroblasts, increased leukocyte infiltration, and epithelial-to-mesenchymal transition support tumor growth and metastasis through the release of pro-angiogenic and immunomodulatory factors [13]. Key players like cancer-associated fibroblasts (CAFs), tumor-associated macrophages (TAMs), and endothelial cells (ECs) present potential targets for therapeutic intervention.

A diverse range of radiotracers is under development and investigation, facilitating personalized therapy planning for individual patients [14,15]. This targeted approach can be utilized for primary imaging and systemic staging, therapeutic interventions (including targeted-biopsy and radiation therapy planning), real-time monitoring of treatment response and recurrence localization. Theranostics, a rapidly evolving field within nuclear medicine, combines diagnostic imaging and targeted therapy using the same molecular agent. This approach allows for the in-vivo mapping of specific molecular targets in tumors through non-invasive and whole-body imaging techniques, such as positron emission tomography (PET) or single-photon emission computed tomography (SPECT), followed by the safe delivery of therapeutic doses of radiation to the same targets. By combining diagnostics and therapy, theranostics enables personalized treatment strategies tailored to the individual patient’s molecular profile. All the discussed tracers in this context have a rather low radiation exposure ranging between 3 and 7.5 mSv depending on the administered amount (e.g., [16]).

Several recent reviews have summarized clinical studies using nuclear molecular imaging in BCa [13,17,18] demonstrating the additional value in this setting. This manuscript focuses on the most recent articles of the last 2–3 years that have evolved based on earlier results. In this review, we discuss various radiotracers and their potential applications in the diagnosis of BCa, as well as provide insights into corresponding radioligand therapy or theranostic approaches. This review addresses the critical need to consolidate and evaluate recent findings in PET and SPECT imaging for BCa, aiming to inform healthcare professionals, as well as researchers, and policymakers about the current landscape and future directions in this rapidly evolving field. It seeks to contribute to the ongoing efforts to improve early detection, treatment personalization, and ultimately, patient outcomes in BCa care. The tracers discussed in this review are summarized in Table 1.

## 2. Principles of Nuclear Medicine in BCa

**Figure 1:**
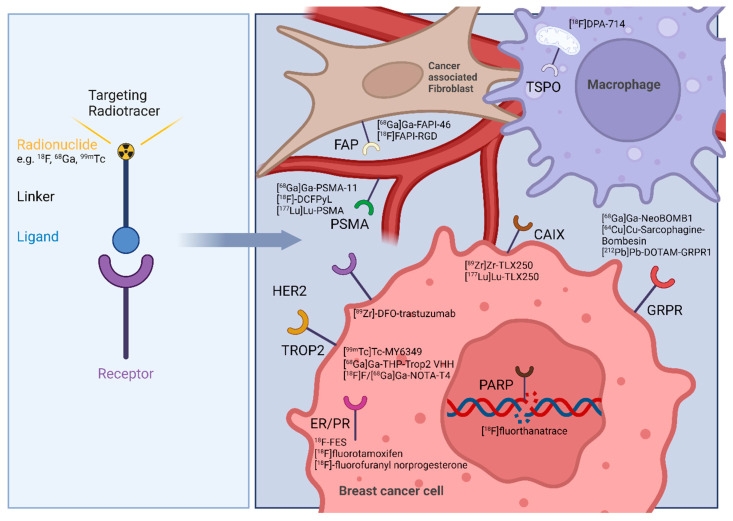
Left: Schematic structure of a targeted radiotracer. Right: breast cancer theranostic targets and their respective radiotracers addressing the BCa cell or tumor microenvironment related cells as cancer associated fibroblasts, and macrophages. Image was created with BioRender.

The heterogeneous nature of BCa subtypes makes the disease even more complex, but advancements in early detection programs, staging accuracy, and imaging techniques have significantly improved survival rates for patients, allowing for better clinical management and treatment planning [39]. When detected early and treated according to guidelines, with a combination of targeted surgery often followed by radiation therapy and, if applicable, hormonal therapy, most cases of BCa are curable [40]. The number of deaths from BCa has been steadily decreasing for decades, even though more women are being diagnosed with BCa [5]. Accurate staging is crucial for optimal patient outcomes. Studies indicate that around 2–10% of BCa are metastatic at diagnosis, highlighting the importance of prompt diagnosis and treatment based on clear signs and symptoms [41]. Regarding specific localized staging investigations employed in the management of BCa, mammography, ultrasound, and computed tomography (CT) scans of the thorax, abdomen, and pelvis are often utilized, with mammography being the mainstay of BCa screening and diagnosis [42]. The standard imaging modality currently used for systemic staging of BCa is SPECT/CT bone scintigraphy according to the National Comprehensive Cancer Network guidelines, with FDG-PET/CT also being considered as an optional standard imaging modality [43]. Additional modalities like magnetic resonance imaging (MRI) are often combined for comprehensive staging assessment [44]. In the realm of BCa diagnosis, radionuclide molecular imaging has shown clear benefits regarding non-invasive tissue characterization compared to conventional anatomical imaging methods like mammography, ultrasound, MRI, and CT, which primarily focus on identifying the structural changes in breast tumors [45]. The use of radiotracers has unlocked new opportunities for visualizing and treating both primary and metastatic cancers. Utilizing PET or SPECT imaging offers insights into the extension of the primary tumor, metastatic status, and molecular expression levels. The variety of BCa phenotypes is the base for the potential of nuclear imaging techniques for precise diagnosis and tailored treatment choices. Radiolabeled ligands targeting hormone receptors (ER, PR, or HER2) offer comprehensive details on the expression status of the receptors and their distribution throughout the body. In the context of TNBC, which lacks ideal cell surface biomarkers for targeted therapy, current research is dedicated to identifying novel probes that can directly target cancer cells, e.g., the poly (ADP-ribose) polymerase enzyme [46], gastrin releasing peptide receptor [47] or the altered glucose metabolism of the cancer cell [48]. By examining the tumor microenvironment of BCa, novel targets have been identified for molecular imaging and theranostics, particularly in the context of invasive or metastatic progression (Figure 1) [49,50]. This review highlights recent advances in radiotracer development, with a particular focus on their clinical application for imaging and treating distinct BCa subtypes while addressing the current gap in the literature regarding subtype-specific, translational approaches in molecular imaging.

## 3. Direct Targeting of Tumor Cells

### 3.1. Radiotracers Targeting the Glucose Metabolism

Tumor cells need to adapt to adverse circumstances like hypoxia and low pH and ensure high proliferation through metabolic reprogramming, where they use aerobic glycolysis as main energy source (Warburg effect) [51].

In molecular imaging, this characteristic feature of increased glucose metabolism is utilized to image a variety of solid tumors. The radiotracer [^18^F]Fluorodeoxyglucose (FDG) uses the glucose analogue FDG, which is coupled to fluoride 18 (^18^F). FDG demonstrates increased uptake in locations of high glucose metabolism like tumors and is visualized by PET.

In 2024, joint EANM/SNMMI guidelines about the role of [^18^F]FDG PET/CT in any type of BCa were published highlighting the importance of this imaging modality in different clinical scenarios [52]. The most important scenarios, presenting good level of evidence and expert agreement, were the initial staging of patients with clinical stage IIB upwards, assessing treatment response and identifying the extent of recurrence.

Robust literature about the usefulness of [^18^F]FDG in BCa has been published and several clinical trials continue to be performed worldwide on this subject [53].

Another study aimed to evaluate the potential of [^18^F]FDG over conventional staging (NCT02751710). Patients with stage IIb or III locally advanced breast cancer were included in the study. Compared to conventional staging, [^18^F]FDG PET/CT detected a higher amount of metastatic lesions [19].

Ongoing trials include NCT04226222.

Although [^18^F]FDG PET/CT is a useful method in the staging of BCa, it is not yet recommended as a first-line diagnostic approach in international guidelines [54].

FDG is the most commonly used radiotracer worldwide and demonstrates increased uptake in most malignancies. Additionally, the overall availability is very good. Still, there are some limitations; for example, [^18^F]FDG is a metabolic marker showing increased glucose consumption in tissue that is not specific to cancer. Infectious or inflammatory tissue is also delineated leading to false positive findings. Moreover, due to its limited spatial resolution, smaller tumor lesions are more difficult to detect [55].

The resulting effective dose is dependent on the applied amount of [^18^F]FDG (between 170 and 250 MBq depending on body weight) and amounts in the range between 3.5 and 7 mSv [16].

### 3.2. Radiotracers Targeting Specific Breast Cancer Subtypes—Hormone Receptor-Positive Tumors

#### 3.2.1. Radiotracers Targeting the Estrogen Receptor

The ER is a member of the steroid receptor family and is expressed in 70–80% of BCa [56]. It plays a key role in the development, progression, and invasion of ER-expressing BC [57]. Two major isoforms are responsible for the growth, differentiation and function of the female reproductive system development, the preservation of bone mass, and the protection of the central nervous system, among other physiologically important processes, namely ERα and ERβ [58,59,60]. While ERβ is expressed in physiologic breast, ovarian, and prostate tissues, ERα is more commonly overexpressed in ER-positive malignancies, with ERβ levels decreasing as the cancer progresses [61]. Therefore, ERα represents the dominant receptor in BCa cells making it a suitable target in molecular imaging of BCa.

The imaging agent 16α-[^18^F]fluoroestradiol ([^18^F]FES) is a PET radiopharmaceutical used for noninvasive imaging of the ER in vivo in patients with metastatic or recurrent BCa. [^18^F]FES binds to both ERα and ERβ at nanomolar levels, but preferentially to ERα by 6.3 fold [62]. In recent years, several studies have been conducted to assess the technical validity, clinical validity and efficacy of [^18^F]FES in the detection and treatment of BCa, which were summarized in several reviews [18]. The product has been approved by the FDA since May 2020 and is marketed under the product name Cerianna [63].

In 2024, the society of Nuclear Medicine and Molecular Imaging released the Procedure Standard/EANM Practice Guideline for Estrogen Receptor Imaging of Patients with BCa, which outlines the usage of [^18^F]FES in different settings [64], namely for (1) determining the ER status when other imaging tests are equivocal or suspicious, (2) assessing ER status in lesions that are difficult to biopsy or when biopsy results are nondiagnostic, (3) considering first-line endocrine therapy at the initial diagnosis of ER-positive metastatic disease, and for (4) considering second-line endocrine therapy after progression of ER-positive metastatic disease.

[^18^F]FES can be used non-invasively to assess the ER status of BCa tumors, offering a significant advantage over invasive immunohistochemistry (IHC). IHC requires a biopsy or surgical tissue sample, which involves invasive procedures that may not always be feasible, particularly in patients with metastatic or recurrent disease. In contrast, [^18^F]FES PET imaging allows whole-body assessment of ER expression, providing a non-invasive alternative that can evaluate multiple metastatic sites simultaneously [65]. This information is important for determining the appropriate treatment approach, as hormone receptor status can influence the choice of hormonal therapy. Additionally, IHC is prone to interpretation inconsistencies. Variability in IHC criteria for ER and PR positivity, with a threshold ranging from 1% to 10% positive cells, leads to inconsistent classification of hormone receptor status [66]. Systematic reviews of IHC for determining HR status have indicated that as many as 20% of current test outcomes globally may be incorrect (either false-negative or false-positive) [67,68]. At the same time, there are still several ongoing studies that investigate the clinical utility of [^18^F]FES PET imaging [69].

The potential use of ER-targeted imaging in the systemic staging of BCa is currently being investigated. To demonstrate the value of [^18^F]FES-PET for systemic staging, prospective studies must show that it is equivalent or superior to these standard imaging modalities. Especially in nearly always ER-positive invasive lobular BCa (ILC), often not all metastases can be detected by an [^18^F]FDG scan. Several studies (NCT01823835, NCT01916122, NCT02316509, NCT02734615, NCT03284957 and NCT03332797) were able to show that in patients with metastatic ILC, [^18^F]FES PET/CT was favorable compared to [^18^F]FDG PET/CT and is still investigated in ongoing clinical trials (NCT04252859, NCT05541367, NCT05982496) [70]. Meanwhile, in another phase II clinical trial investigating the efficacy of stereotactic body radiation therapy in the treatment of oligoprogressive ER-positive metastatic BCa, [^18^F]FES is being used for treatment monitoring (NCT06260033).

It has also been shown that the use of [^18^F]FES is helpful in determining the biologically effective dose for new ER-targeted therapeutics in early clinical trials [71,72,73,74,75]. While some studies have already been completed or terminated (NCT01377324, NCT02650817), one is still ongoing (NCT01273168).

Other studies have demonstrated the prognostic and predictive properties of [^18^F]FES for various hormone therapies. Studies have already demonstrated that [^18^F]FES is able to select patients that will benefit from hormonal therapies [76,77]. Currently, [^18^F]FES is under investigation to optimize tamoxifen dosing (NCT04174352), guide therapy in ER-positive metastatic BCa (NCT05068726, NCT05486182), assess ER expression in metastases with ER-negative primary tumor (NCT06344767), and evaluate axillary lymph node metastasis in ER-positive BCa (NCT06695039). While the clinical utility of [^18^F]FES is well established, several technical limitations should be considered. False-negative results are among the most significant, often occurring in lesions within or near areas of high physiological uptake such as the liver, peritoneal metastases near the bowel, bowel metastases, and uterine metastases, making detection challenging. False positives, though less common, can appear in non-breast pathologies like uterine leiomyoma, meningioma, atelectasis, pneumonitis, and interstitial lung disease. Notably, [^18^F]FES uptake in the lungs due to atelectasis or radiation-induced changes can be misinterpreted as ER-positive disease because lung tissue has some estrogen-binding properties.

Another major limitation is the assessment of liver metastases. The liver metabolizes [^18^F]FES, leading to high background uptake in the liver parenchyma, which can obscure metastatic lesions and reduce sensitivity. Improving the detection of liver metastases is an ongoing research focus. Delaying the imaging several hours after injection may improve visualization of hepatic lesions due to [^18^F]FES washout over time.

Finally, the use of [^18^F]FES PET/CT is limited by ER-antagonist therapies, which require long washout periods—about 8 weeks for tamoxifen and up to 28 weeks for fulvestrant—making timely imaging difficult in some clinical scenarios [78,79].

In addition to the originally designed FES, other derivatives have been developed to increase the stability and affinity of the tracer, as [^18^F]FES has slow blood clearance and rapid metabolism [80,81]. After 4-fluoro-11β-methoxy-16α-18F-fluoroestradiol ([^18^F]F-4FMFES) had shown good preclinical results [82,83,84], a clinical phase I showed that [^18^F]F-4FMFES was highly taken up in the uterus of pre- and postmenopausal women, has a faster blood clearance and a lower uptake in most non-specific organs than observed with [^18^F]FES [85]. Some preliminary results of the phase II clinical trial, investigating the evaluation of the diagnostic, prognostic and follow-up potential of [^18^F]F-4FMFES PET imaging for ER-positive advanced BCas, have shown that [^18^F]F-4FMFES PET achieves a lower non-specific signal and better tumor contrast than [^18^F]FES PET, resulting in improved diagnostic confidence and lower false-negative rates (NCT04824014) [20].

Even though the tracer is FDA-approved and has been widely investigated in clinical studies, it has several drawbacks. Its positive predictive value is limited; a positive result does not always indicate that a tumor will respond to endocrine therapy due to nonfunctional ER or other resistance mechanisms. Additionally, FES imaging may not effectively detect tumors with low ER expression or those that are small in size. Rapid metabolism and clearance of FES in the body can further complicate accurate measurements, while uptake variability between different tumor sites highlights its dependence on tumor biology and heterogeneity. Lastly, the inability of FES to provide information about the functionality of ER underscores the need for complementary biomarkers and imaging tools [86,87].

A further possible method for PET imaging of ER-positive BCa is the radiolabeling of endocrine drugs. The most commonly used endocrine drug tamoxifen was previously labeled with iodine-123 and fluorine-18. While only ER-positive and PR-positive tumors could be detected with [^123^I]iodotamoxifen, but not ER-positive and PR-negative as well as tumors negative for both markers [88,89], a preliminary study with [^18^F]fluorotamoxifen showed that [^18^F]FTX PET imaging can be used to predict the response to tamoxifen therapy [90]. However, [^18^F]FTX is not yet widely available and has so far been evaluated only in small patient cohorts. Moreover, ongoing endocrine therapy may interfere with tracer uptake, introducing challenges in image interpretation. Inconsistent correlations between [^18^F]FTX uptake and ER expression, as well as discordance between uptake and therapeutic response, further underscore the need for larger, prospective studies to validate its specificity, reproducibility, and clinical utility.

#### 3.2.2. Radiotracers Targeting the Progesterone Receptor

The progesterone receptor is an estrogen-related protein with two predominant isoforms, PR-A and PR-B. However, as knowledge of PR is incomplete compared to the other BCa hormone receptors, only limited progress has been made in PR imaging [91]. It is known that the measurement of changes in PR protein expression in metastatic BCa can serve as an indicator of the functional activity of the ER and the endocrine therapy response [92]. Thus, PR-targeted imaging may be useful to predict response to ER-targeted endocrine therapy and might be a predictive biomarker for PR-targeted therapy. Currently, clinical trials evaluating PR imaging in breast cancer are limited, but there are a few studies in progress.

The most studied PR-targeted radiopharmaceutical is the steroidal progestin [^18^F]-fluorofuranyl norprogesterone ([^18^F]FFNP). A first-in-human feasibility study (NCT00968409) showed drug and organ radiation dose safety among BCa patients and a correlation between tumoral [^18^F]FFNP uptake and PR expression scores based on IHC [93]. In a following phase II clinical trial (NCT02455453), it was shown that the change in [^18^F]FFNP uptake in a tumor after estradiol provocation is highly predictive of response to endocrine therapy in women with ER-positive BCa [94]. Currently, the tracer is investigated in three different phase II clinical trials for assessing PR expression in invasive BCa (NCT03212170), predicting predict response to presurgical endocrine therapy (NCT06086704), and identifying biomarkers in ER- and PR-positive metastatic lobular BCa patients starting a new endocrine therapy (NCT06067503). Despite promising results, [^18^F]FFNP uptake is highly dependent on ER signaling, and PR expression can be suppressed in postmenopausal patients or those receiving aromatase inhibitors, potentially causing false-negative findings. Additionally, physiological uptake in PR-expressing organs such as the uterus and ovaries can complicate image interpretation [94]. Another tracer for HR-positive and HER2-negative BCa and triple negative BCa, [^68^Ga]Ga-NNS309, is being investigated in a phase I clinical trial (NCT06562192). However, there are no published results for any of the clinical studies.

#### 3.2.3. Radiotracers Targeting the Human Epidermal Growth Factor Receptor 2

The human epidermal growth factor receptor 2 (HER2, also known as ErbB2) is one of the most extensively studied receptors for BCa. Numerous studies with radiolabeled monoclonal antibodies targeting HER2 have been conducted, which have been discussed in detail in previous reviews [17,18]. The Df-Bz-NCS conjugated zirconium-89 labeled trastuzumab is one of the most investigated. Previous studies have shown that ^89^Zr-trastuzumab supports clinical decision making when HER2 status could not be determined by standard procedures (bone scan, ^18^F-FDG PET, CT and biopsy) and is able to determine tumor heterogeneity (NCT01832051, NCT01565200) [95,96]. However, a further phase I clinical trial showed that the dosimetry of this tracer was not satisfactory, so the tracer was produced by site-specific conjugation of the new chelator, p-SCN-Bn-Desferrioxamine (SCN-Bn-DFO) [97,98], on enzymatically modified glycans of trastuzumab. This tracer, ^89^Zr-DFO-trastuzumab, is currently being tested in a phase I clinical trial in patients with HER2-positive breast or gastric cancer (NCT05955833).

Another tracer utilizes radioactive copper with a 1,4,7,10-tetraazacyclododecane-1,4,7,10-tetraacetic acid (DOTA) chelate, coupled to the trastuzumab antibody core. Several studies with this tracer have shown that ^64^Cu-DOTA-trastuzumab PET imaging could detect primary HER2-positive BCa and metastatic lesions [99]. It was also shown in a phase I clinical trial with 40 patients that the tracer is able to detect metastatic brain lesions [100]. This is now being investigated in a phase IV clinical trial to evaluate if the tumor uptake may predict the response to trastuzumab deruxtecan (NCT05376878).

Recent clinical investigations have focused on utilizing engineered scaffold proteins, including affibody molecules, designed ankyrin repeat proteins (DARPins), and albumin-binding domain derived affinity proteins (ADAPTs). These engineered scaffold proteins have a robust protein framework in common, which is based on different scaffolds. With molecular weights ranging from 4 to 20 kDa, they have short residence times in the bloodstream, enabling imaging within hours after application. Affibodies are small molecules approximately 7 kDa in size that are based on an immunoglobulin scaffold [17]. They are characterized by their fast kinetics, high affinity to receptors and short circulation time, which is why affibody molecules are very well suited for use in molecular imaging. Clinical studies have shown that radiolabeled HER2 Affibody can provide highly specific and sensitive PET or SPECT imaging by labeling with ^68^Ga [101,102,103], ^99m^Tc [104,105], or ^111^In [106]. The most investigated affibody in clinical trials is ZHER2:2891 (ABY-025) (NCT01216033, NCT01858116, NCT02095210, NCT03655353). The affibody has a site-specific labeling via the chelator DOTA conjugated to a C-terminal cysteine [107]. The [^68^Ga]Ga-ABY-025 affibody has demonstrated high stability, fast blood clearance and high-contrast imaging in HER2 expressing tumors that enabled the discrimination between BCa metastases with high and low HER2 expression levels [101,102]. Currently, the tracer is being investigated in a phase II clinical trial (NCT05619016) in patients with HER2-low metastatic BCa. The aim is to enhance the identification of patients with solid tumors who would benefit from effective treatment with HER2-targeted drugs. The initial results showed that imaging with [^68^Ga]Ga-ABY-025 was feasible and safe. The observed diversity in [^68^Ga]Ga-ABY-025 uptake, both within and among individuals, indicates that this imaging technique could be used to non-invasively evaluate disease heterogeneity [21].

Currently, the same affibody conjugated with a different chelator is being investigated in a phase I clinical trial (NCT05411432). The tracer [^68^Ga]Ga-NOTA-MVK-ZHER_2:2891_ has been investigated in seven healthy volunteers for biodistribution and dosimetric studies and compared with the scans with the control ligand [^68^Ga]Ga-NOTA-Z_HER2:2891_ performed one week apart. The initial results showed that the kidney uptake of the MVK ligand was, with an SUV_mean_ of 34.3 and 45.8, respectively, significantly lower than that of the control ligand at 1 h p.i., while the uptakes of the two ligands in the other organs showed negligible differences. Urine samples from healthy volunteers administered the MVK ligand displayed the presence of the enzymolysis fragment [^68^Ga]Ga-NOTA-Met-OH, affirming the effectiveness of the enzymolysis clearance method in humans. The PET/CT study of patients showed that HER2-positive lesions exhibited SUV_max_ values ranging from 9.4 to 21, whereas HER2-negative lesions displayed values between 2.7 and 6.2. These findings suggest that the alteration involving MVK did not interfere with Z_HER2:2891_ structure’s capacity to bind to HER2 and further research is warranted [22].

Single-domain antibodies (sdAb, also known as nanobodies) are the smallest antibody fragment derived from camelid heavy chain-only antibodies. Their small size of 12-15 kDa enables them to penetrate tumor tissue and bind to the antigen with high specificity. Two sdAbs that have been generated include ^99m^Tc-MIRC213 and ^99m^Tc-MIRC208. The latter has been investigated in a clinical trial (NCT04591652) to determine the status of HER2 in cancer patients. The initial results indicate that primary and metastatic HER2-positive lesions of patients were clearly visualized by ^99m^Tc-MIRC208 SPECT at 2 h following injection [23]. ^99m^Tc-MIRC213 is currently being investigated in an early phase I clinical trial (NCT05622240) to detect HER2-positive BCa and to compare its diagnostic value with routine IHC staining [23,24]. Furthermore, ^68^Ga-ABS011 and ^177^Lu-RAD202 are currently being investigated in clinical trials (NCT06369831, NCT06824155).

The metabolic effects of endocrine treatment in HER2-positive breast cancer can be monitored with [^18^F]FDG. The recent PHERGain study assessed the impact of trastuzumab and pertuzumab on primary tumors and axillary lymph nodes and their prognostic value for a pathological complete response (NCT03161353). Cohort A received trastuzumab and pertuzumab with docetaxel and carboplatin, whereas cohort B received trastuzumab and pertuzumab with endocrine therapy instead of chemotherapy. [^18^F]FDG scans were performed at the start of the treatment and after two cycles of therapy. The results demonstrate that [^18^F]FDG was able to identify HER2-positive patients, which most likely can benefit from chemotherapy-free treatment using only the HER2-targeted blockade [25].

Despite the promising developments in HER2-targeted molecular imaging, several limitations must be considered. One important challenge is the variation in pharmacokinetics among different tracer types: while full-length monoclonal antibodies have slow tissue penetration and long circulation times, smaller constructs such as affibodies, DARPins, and nanobodies demonstrate rapid blood clearance and earlier optimal imaging timepoints—but may also result in high renal uptake, potentially limiting sensitivity for detecting lesions near the urinary tract [17].

#### 3.2.4. Radiotracers Targeting the Gastrin Releasing Peptide Receptor

Another protein that is upregulated in different cancers, including hormone receptor-positive BCa as well as TNBC, is the gastrin-releasing peptide receptor (GRPR) [47]. In 76% of primary BCa samples, overexpression of GRPR was detected by IHC. A strong correlation was observed between GRPR and ER overexpression, consistent with previous research findings showing high GRPR levels in 83% of ER-positive tumors and 12% of ER-negative tumors. High expression of GRPR was not only observed in primary tumors of the luminal A and B BCa subtypes (86% and 70%, respectively) but also in 95% of the analyzed metastatic lymph nodes [108]. D’Onofrio et al. reviewed in detail the actual landscape of GRPR tracers in preclinical as well as clinical imaging and therapeutic studies for BCa [109].

[^68^Ga]Ga-NeoBOMB1 is a novel DOTA-coupled high-affinity GRPR antagonist for PET imaging, which has already been evaluated within an initial “MITIGATE-NeoBOMB1” phase I/IIa clinical trial in patients with (oligo)metastatic gastrointestinal stromal tumors (EudraCT 2016-002053-38, ref. [110]). Based on the positive outcome of this study, in which the tracer was well tolerated, and variable tumor-specific uptake was observed, the targeting properties of [^68^Ga]Ga-NeoBOMB1 in patients with other malignancies known to overexpress GRPR were investigated (NCT03724253, ref. [26]). Within the “NeoFIND” open-label, multicenter, phase II study, starting in 2018, [^68^Ga]Ga-NeoBOMB1 was evaluated in 19 patients, including 5 patients with BCa. Results from 2020 indicate that two BC patients who were enrolled in the dosimetry subgroup had effective whole-body doses of 0.0203 and 0.0151 mSv/MBq. These results underline previous dosimetry data obtained by the administration of the same tracer to patients with gastrointestinal stromal tumors of the MITIGATE-NeoBOMB1 study. Even though the tracer was well tolerated and detected different tumor types, tumor-specific uptake was variable and difficulties in enrolling patients for a diagnostic study led to premature termination of the study. Nonetheless, a follow-up therapeutic phase I/IIa study named “NeoRay” evaluated the safety, tolerability, pharmacokinetics, distribution, radiation dosimetry and anti-tumor activity of [^177^Lu]Lu-NeoBOMB1, amongst other cancers in BCa (HR positive, HER2 negative and HER2 low) (NCT03872778, ref. [27]).

A phase I study from 2022 in Australia assessed the safety and potential of [^64^Cu]Cu-Sarcophagine-Bombesin ([^64^Cu]Cu-SAR-BBN) PET/CT for re-staging in seven patients with metastatic HR-positive expression and HER2-negative expression [28]. No adverse events were reported. Staging with conventional imaging ([^18^F]FDG, bone scan and diagnostic CT) was carried out within three weeks before administration of the tracer. Six of seven patients were positive on [^18^F]FDG imaging, while five of seven patients were positive on [^64^Cu]Cu-SAR-BBN imaging. Four patients were positive with both tracers and higher uptake and higher avidity were observed with [^64^Cu]Cu-SAR-BBNGRPR. One patient was [^64^Cu]Cu-SAR-BBN-positive/[^18^F]F-FDG-negative. Further investigation is warranted.

In a recent phase I imaging study conducted in 2023, researchers from the Tomsk National Research Medical Center investigated the biodistribution, dosimetry, safety, and tolerability of [^99m^Tc]Tc-DB8 in a cohort comprising five patients with prostate cancer and five patients with BCa. Additionally, the study endeavors to correlate the [^99m^Tc]Tc-DB8 SPECT imaging findings with those obtained from CT, MRI, ultrasound examinations, and IHC analyses. The results of this investigation are pending publication (NCT05940298).

In 2022, a therapeutic GRPR-targeting study (phase I) was initiated, which evaluated the tracer [^212^Pb]Pb-DOTAM-GRPR1 for safety and effectiveness in patients with various GRPR-expressing tumors, including BCa (NCT05283330). After determination of the recommended Multiple Ascending Dose (MAD) dose, the subjects were treated with up to four cycles of the alpha emitting radiotracer, administered every eight weeks.

Using [^177^Lu]Lu-NeoB, a therapeutic tracer targeting GRPR, Novartis has initiated two clinical studies (NCT05870579, NCT06247995) in adult female patients with ER-positive, HER2-negative, GRPR-positive advanced or metastatic BCa. To date, no results from these trials have been published.

With regard to specificity and off-target binding, GRPR is also physiologically expressed in several non-tumor tissues, including the pancreas, gastrointestinal tract, and normal breast tissue [111]. In particular, uptake in normal breast tissue can lead to increased background signal, thereby reducing tumor-to-background contrast and complicating image interpretation. Additionally, accurate quantification of tracer uptake can be challenging due to spill-in from adjacent organs and partial volume effects, especially in small lesions, making it difficult to reliably detect low-expressing tumors or assess treatment response over time.

### 3.3. Radiotracers Targeting Hormone Receptor Negative Breast Cancer Subtypes

In contrast to the hormone receptor-positive BCa phenotypes mentioned earlier, hormone receptor negative BCa types, e.g., TNBC, do not express receptors necessary for targeted therapies. However, ongoing research is focused on discovering new targets and creating imaging and theranostic probes to treat this aggressive subtype of receptor negative BCa [112].

#### 3.3.1. Radiotracers Targeting the Poly (ADP-Ribose) Polymerase Inhibitors

One prominent target for TNBC imaging and treatment is the poly (ADP-ribose) polymerase (PARP) enzyme, which plays a role in DNA repair [113]. Inhibitors of PARP (PARPi), such as olaparib and talazoparib, have shown efficacy in TNBC patients with BRCA mutations, as these tumors are more sensitive to PARP inhibition due to their impaired DNA repair mechanisms [114]. Recently, several PET radiotracers have been developed to image PARP-1 expression levels in cancer patients. Puentes et al. reviewed the development and evaluation of 10 PARP-1 targeting radiotracers reported in the literature over the past 5 years [46]. These include [^18^F]PARPi, [^18^F]fluorthanatrace (FTT), and [^18^F]olaparib. Among these, only FTT has been utilized in translational imaging studies across various tumor types such as BCa, hepatocellular carcinoma, and ovarian cancer (NCT03604315, refs. [29,115,116]). Lee et al. summarized the development of FTT as a PET tracer from bench to clinical phase II trials [117]. McDonald et al. started with a prospective nonrandomized clinical trial, where 30 patients have been included to investigate the correlation of FTT uptake across a range of BCa phenotypes including ER-positive, HER2-positive, and TNBC. Another study indicated that tracer binding was heterogenous across and within different BCa phenotypes, including germline or tumor mutations in BRCA-related genes (NCT03083288, ref. [30]). As a follow-up study, patients with metastatic BCa have been recruited. FTT uptake was blocked after receiving PARPi therapy in known sites of disease, indicating the specificity as a biomarker for PARPi treatment (NCT03846167, ref. [29]). In a phase II clinical trial, which is currently running, the diagnostic potential of FTT will be further evaluated (NCT05226663). In this multicenter clinical study, Washington University in St Louis, University of Pennsylvania, and MD Anderson Cancer Center will recruit a total of 36 patients with primary invasive BCas with tumor size measuring at least 1 cm and undergoing upfront surgery.

To date, PARP-targeted radiotracers under clinical investigation have been limited to imaging applications. This is largely due to the structural constraints of PARPi, which do not tolerate modification with conventional chelators without compromising their binding affinity to the PARP enzyme. As a result, radiolabeling has primarily relied on radiohalogens such as fluorine-18. However, for theranostic applications, attempts to substitute fluorine with larger halogens like iodine-125 or iodine-131 may reduce radiotracer stability [118], potentially diminishing target binding and increasing off-target toxicity due to non-specific accumulation.

#### 3.3.2. Radiotracers Targeting Hypoxia-Related Factors

With the rapid growth of solid tumors, they often outpace the development of new blood vessels. This leads to a decrease in oxygen levels from the normal range of 2–9% to less than 2%, creating a condition of acute hypoxia. Hypoxia is dependent on perfusion and arises from alterations in blood flow from nearby vessels, causing fluctuations in oxygen diffusion [119]. The rapid growth of aggressive tumors like TNBC stimulates angiogenesis to counteract nutrient deprivation. Tumor-induced angiogenesis manifests as a disorganized vasculature, leading to greater distances between blood vessels and adjacent capillaries resulting in chronic hypoxia, fostering various pathways for tumor expansion [120].

Girentuximab (cG250) is a chimeric monoclonal antibody that targets carbonic anhydrase IX (CAIX), which is a cell surface antigen expressed in various cancers, such as clear cell renal cell carcinoma (ccRCC), urothelial carcinoma, and TNBC. CAIX is strongly upregulated in response to hypoxia in different types of solid tumors and is a strong predictor of poor outcome in patients with TNBC [121,122]. Within the phase II OPALESCENCE study, girentuximab is labeled with ^89^Zr ([^89^Zr]Zr-TLX250) for PET imaging of metastatic TNBC patients (NCT04758780). Telix Pharma published the results of the pilot prospective study of twelve metastatic patients that demonstrated the expression of CAIX in TNBC and effective targeting with [^89^Zr]Zr-TLX250 [31]. Expression and targeting of CAIX in lesions in the breast, skin, adrenal gland, and brain was 100% and expression in lymph nodes and bone was 88% and 91.9%, respectively. Overall, the tracer was safe and well tolerated in this cohort of patients. Because of these promising results Telix Pharma aims to broaden its theranostic CAIX program with prospective utilization in ^177^Lu and ^225^Ac therapies. In May 2023, the open label, single-arm, multicenter dose escalation and dose expansion phase I study was initiated evaluating different combinations of three radioactive dose levels of therapeutic [^177^Lu]Lu-TLX250 with three different doses of peposertib in patients with CAIX-expressing solid tumors (NCT05868174). Whereas cohort A only included metastatic or non-resectable ccRCC, cohort B included 20 patients with CAIX-positive solid tumors (excluding RCC). The number of BCa patients included in Cohort B remains unknown.

Another tracer for imaging hypoxia is the PET tracer, fluoromisonidazole ([^18^F]F-FMISO). It quantitatively measures hypoxia levels intratumorally, as it is retained by irreversible binding to the thiol-rich metabolic proteins at rates that are inversely proportional to oxygen concentration [123]. While [^18^F]F-FMISO has been widely employed in clinical trials for glioblastoma, its broad adoption has been limited due to its reduced sensitivity in detecting less hypoxic tumor areas. Nonetheless, this feature could prove advantages in immunotherapy, as profound hypoxia is often linked to an immunosuppressive tumor microenvironment [124,125]. In 2025, an early phase I clinical study will investigate the utility of [^18^F]F-FMISO in patients diagnosed with TNBC (stage II-IV disease) to monitor and predict the effect of immunotherapy (NCT04861077). If the obtained data prove to be promising, it will serve as the foundation for designing larger-scale trials.

However, [^18^F]F-FMISO exhibits slow clearance from normoxic tissues [126], which can lead to low tumor-to-background ratios—especially in regions with only mild hypoxia. This limitation reduces image contrast and may impair the detection of less hypoxic tumor areas. Moreover, the tracer’s pharmacokinetics necessitate delayed imaging, typically several hours post-injection, which poses logistical challenges and reduces efficiency in routine clinical workflows.

#### 3.3.3. Radiotracers Targeting CXCR4

CXCR4 is a chemokine receptor that is often overexpressed in invasive BCa and has been demonstrated to play a significant role in the signaling pathways associated with metastasis [127]. In cancer, the expression of CXCR4 and its activation by the endogenous ligand CXCL12 are crucial factors that initiate tumor growth, progression, invasiveness, and metastasis [128].

In the study conducted by Vag et al. (2018), a total of eighteen patients were evaluated for CXCR4-targeted PET imaging using [^68^Ga]Ga-Pentixafor [32]. Among these, thirteen patients were newly diagnosed with BCa, four patients had recurrent disease following initial treatment, and one patient presented with axillary lymph node metastasis of unknown primary origin. This diverse cohort allowed for a comprehensive assessment of the diagnostic capabilities of CXCR4-targeted imaging in various stages of BCa. Out of the thirteen primary BCa assessed, nine were visually identifiable on [^68^Ga]Ga-Pentixafor PET images. The lesions that were not visually detectable included both instances of invasive lobular carcinoma and two cases of invasive carcinoma of no special type, which were triple-negative and lacked hormone receptor and HER2 expression. In contrast, all five cases of metastases from recurrent BCa and unknown primary cancer were visually identifiable. [^18^F]-FDG PET exhibited higher SUVmax values across all patients when compared to [^68^Ga]Ga-Pentixafor PET.

In 2023, Watts and colleagues investigated the use of [^68^Ga]Ga-Pentixafor PET/CT as an imaging modality for patients with BCa, comparing its effectiveness with [^18^F]-FDG PET/CT [33]. The study included seven patients, with both [^18^F]FDG and [^68^Ga]Ga-Pentixafor PET/CT performed on three of these individuals. Increased uptake of [^68^Ga]Ga-Pentixafor PET/CT was observed in both primary and metastatic lesions (including lymph nodes and skeletal sites) in four out of the seven patients; two of these patients had previously undergone surgery, while the other two had not had any surgical intervention. No evidence of disease was found in three of the seven patients. In a direct comparison of [^18^F]FDG and [^68^Ga]Ga-Pentixafor in the three patients, both tracers identified primary and metastatic lesions, but the SUVmax values for most of the lesions were higher with [^18^F]FDG compared to [^68^Ga]Ga-Pentixafor.

In 2023, Chonco and colleagues, similar to Watts and their team, aimed to evaluate the diagnostic efficacy of CXCR4-targeted PET imaging in BCa patients using [^68^Ga]Ga-Pentixafor [34]. A total of thirty patients with either newly diagnosed (*n* = 29) or previously treated (*n* = 1) locally advanced BCa underwent [^68^Ga]Ga-Pentixafor PET/CT scans. Among these patients, 14 out of 30 (47%) had TNBC, 12 out of 30 (40%) had ER-positive tumors, and 10 out of 30 (33%) were classified as Luminal B subtype. The results showed that [^68^Ga]Ga-Pentixafor PET/CT was visually positive in all cases; however, [^18^F]-FDG exhibited higher SUVmax values across all patients compared to [^68^Ga]Ga-Pentixafor PET. Notably, there was no correlation between the SUVmax obtained from [^68^Ga]Ga-Pentixafor PET and prognostic factors, including molecular subtypes, ER, PR, HER2 status, tumor grade, or proliferation index. Interestingly, TNBC demonstrated more avid accumulation of [^68^Ga]Ga-Pentixafor compared to Luminal A and B subtypes.

#### 3.3.4. Additional Radiolabeled Antibodies Targeting Tumor Cells

In addition to these targeted approaches, researchers are also investigating the use of immunotherapy combined with radionuclides for TNBC. Monoclonal antibodies (mAbs) have the ability to target tumor cell antigens specifically. This unique feature has led to their use in the targeted delivery of radioisotopes to tumor sites (imaging and radioimmunotherapy (RIT)). The emergence of immunotherapy, specifically immune checkpoint inhibitors (ICIs), has transformed the treatment of solid tumor malignancies. In BCa, the most compelling evidence for ICI therapy pertains to TNBC. Preclinical investigations have indicated increased antitumor immune responses in TNBC patients treated with ICIs. Initial clinical studies explored the efficacy of ICI monotherapy in metastatic TNBC patients, showing promising outcomes, especially in the frontline setting and in individuals with high levels of programmed cell death 1 (PD-1) or programmed cell death ligand 1 (PD-L1) expression. Subsequent trials have assessed the combination of ICIs with standard chemotherapy to bolster the host immune response [129].

Currently, the gold standard biomarker for selecting ICI therapy is the measurement of PD-L1 protein expression through IHC. This method has several drawbacks, including inter- and intra-tumoral heterogeneity in PD-L1 expression, as well as the requirement for invasive procedures to acquire material for analysis. In 2023, the Karolinska University Hospital started an observational clinical trial to improve precision medicine through more refined therapy selection for BCa patients who are candidates for ICI therapy (NCT05742269). Atezolizumab is a humanized monoclonal antibody of the IgG1κ type that specifically binds to PD-L1. It inhibits the interaction between PD-L1 and the PD-1 receptor. By binding to PD-L1, atezolizumab stimulates the activation and proliferation of cytotoxic CD8+ T cells, leading to enhancement or restoration of the tumor-directed T cell response [130]. In a recently initiated study, PET/CT imaging with the radiotracer [^89^Zr]Zr-atezolizumab, visualizing PD-L1 expression in the whole body, was evaluated as a better predictive biomarker for a selection of patients that could benefit from ICI. The study compares the level of statistical agreement between PD-L1 status on IHC and PET/CT with [^89^Zr]Zr-atezolizumab.

A further promising target in radio-immunotherapy is the trophoblast cell surface antigen 2 (TROP2), also known as tumor-associated calcium signal transducer 2. TROP2 is a cell surface glycoprotein that functions as a transmembrane transducer of intracellular calcium signaling. While it is present in numerous normal tissues, TROP2 is notably upregulated in various cancers including BCa [131,132]. In 2023, three clinical trials dealing with TROP2 targeting radio-conjugated antibodies for imaging were started. In September 2023, the Peking Union Medical College Hospital started to investigate the tracer [^99m^Tc]Tc-MY6349 in TNBC patients (NCT06104085). The study aims to monitor the expression level of TROP2 in patients with metastatic tumors by SPECT/CT imaging. Subsequently, [^18^F]FDG PET/CT imaging is being performed to compare and detect the distribution of primary tumors and metastases in TNBC patients. This study analyzes the heterogeneity of TROP2 expression levels within the primary tumor and the heterogeneity of expression levels in systemic metastases, thereby providing a base for testing whether the patient is suitable for subsequent treatment and conducive to the formulation of subsequent treatment plans.

In another study initiated in October 2023, the antibody-drug conjugate [^68^Ga]Ga-THP-Trop2 VHH is being tested as a non-invasive approach to detect the TROP2 expression of tumor lesions in patients with solid tumors and to identify patients that would benefit from TROP2 targeting antibody–drug conjugate treatment (NCT06188468). These results will be compared to the standard of care imaging for each type of tumor ([^18^F]F-FDG, [^68^Ga]Ga-PSMA, or [^68^Ga]Ga-DOTATATE PET/CT) to evaluate the diagnostic accuracy.

[^18^F]F/[^68^Ga]Ga-NOTA-T4 is a radiolabeled antibody targeting TROP2 that is currently under clinical investigation (NCT06203574). The study started in December 2023 and deals with the investigation of the diagnostic efficacy and safety of [^18^F]F/[^68^Ga]Ga-NOTA-T4 in pancreatic cancer, BCa, or head and neck malignant tumors.

A further potential target for radio-immunotherapy is the insulin-like growth factor-1 receptor (IGF-1R). In 2019, Fusion Pharmaceuticals Inc. started a clinical trial investigating the imaging and therapy potential of [^111^In]In-FPI-1547 and [^225^Ac]Ac-FPI-1434, respectively. Both tracers consist of an IGF-1R-targeting humanized monoclonal antibody, a bifunctional chelator, and a radionuclide (NCT03746431). This is a first-in-human phase I/II, non-randomized, multi-center, open-label clinical study designed to investigate safety, tolerability, and preliminary anti-tumor activity of [^225^Ac]Ac-FPI-1434 in patients with solid tumors that demonstrate uptake of [^111^In]In-FPI-1547, and to establish the maximum tolerated dose (MTD) and/or the recommended phase II dose of repeated doses of [^225^Ac]Ac-FPI-1434 injections. Results are available for 13 patients with solid tumors (not further clarified) from the single dose administration portion of [^111^In]In-FPI-1547 of the study. All 13 (100%) demonstrated avidity in at least one lesion and all were eligible to receive [^225^Ac]Ac-FPI-1434 based on dosimetry. Twelve (92%) patients received at least one therapeutic administration of [^225^Ac]Ac-FPI-1434 and demonstrated a manageable safety profile with no drug-related serious adverse events and/or dose limiting toxicity [35].

Antibody-based tracers can provoke immune responses and often display slow systemic clearance due to their large molecular size, which restricts renal excretion by limiting passage through the glomerular filtration barrier [133]. As a result, background activity may remain elevated, reducing overall imaging contrast. These limitations can be addressed by humanizing antibody structures or employing smaller peptide-based tracers, which offer faster clearance and improved biodistribution profiles.

## 4. Radiotracers Targeting the Tumor Microenvironment

The TME consists of different cell types like fibroblasts, endothelial cells, and immune cells. Complex signaling mechanisms between the TME and the tumor cells influence tumorgenesis, disease progression and therapy resistance [134]. Low pH and oxygen levels and the presence of specific soluble factors are further characteristics of the TME [135]. CAFs and TAMs secrete factors, which promote epithelial-to-mesenchymal transition in cancer cells. This facilitates the invasion in secondary organs and promotes stemness [136].

### 4.1. Fibroblast Activation Protein (FAP)

CAFs are the most abundant cell fraction in BCa promoting lung metastasis and also inducing radioresistance of tumor cells [137]. Compared to normal fibroblasts, they exhibit a different phenotype and differences in gene or protein expression, e.g., overexpression of FAP [134], which is a rising target for the development of radiolabeled pharmaceuticals. Due to its limited presence in normal tissues, FAP is an attractive target for imaging and radiotherapy. Recently, PET/CT imaging using FAP inhibitors (FAPIs) has gained significance for diagnosing and potentially treating various cancers [138].

The developments regarding FAPI labeling are very promising and warranted. Some studies have directly compared FAPI and FDG PET/CT in the same patient with lobular BCa and showed better results compared to FAPI [37]. Furthermore, recent research comparing FAPI and FDG in the same patient also demonstrated the advantages of FAPI, suggesting it should be considered in patients with early-stage IIA [130,139,140].

A recent study aims to compare the diagnostic potential of [^18^F]FDG versus [^68^Ga]Ga-FAPI-46 in ER-positive breast cancer (NCT06335069). Usually, imaging with [^18^F]FDG is recommended for locally advanced breast cancer; however, the primary tumor uptake in ER-positive lesions is significantly less than in HER2-positve or TNBC. Therefore, it will be evaluated if FAP-targeted imaging could be a more suitable choice for the diagnosis of ER-positive tumors. The potential of [^68^Ga]Ga-FAPI-46 for BCa imaging has already been demonstrated [37]. Patients with newly diagnosed BCa were enrolled in this study. Compared to [^18^F]FDG, [^68^Ga]Ga-FAPI-46 was able to identify axillary lymph node metastases more frequently and showed a significantly higher uptake in primary tumors, which highlights the potential as an alternative for [^18^F]FDG. However, the results are not surprising, as [^18^F]FDG is not recommended to stage the axilla. Despite promising results, [^68^Ga]Ga-FAPI-46 also demonstrated limited availability due to the short half-life of ^68^Ga and dependency on generator-based production. Moreover, increased uptake in inflammatory or fibrotic tissue may reduce specificity, and the interpretation of FAPI PET images still lacks standardization in clinical guidelines [141].

FAPI-labeled tracers offer the advantage that DOTA as the chelator can also be coupled to several therapeutic nuclides and therefore allow a theragnostic approach. Still, FAPI tracers are not specific to cancer, as they accumulate in postsurgical wound healing and other diseases, which involve tissue remodeling. The estimated radiation exposures in this study of [^68^Ga]Ga-FAPI-2 and [^68^Ga]Ga-FAPI-4 were 1.4 and 1.8 mSv/100 Mbq [142]. Moreover, the production of [^68^Ga]Ga-FAPI is not dependent on the availability of a cyclotron due to the availability of Ge/^68^Ga generators.

Ongoing trials without published results include NCT05574907, NCT06225505, NCT06175390, and NCT05976620.

### 4.2. Endothelial Cells

Tumor growth and metastasis are dependent on the availability of oxygen and nutrients. These are supplied via the tumor-associated vasculature. These blood vessels differ from the healthy vasculature not only regarding their structure and morphology, but also in their genetic expression patterns. Several newly identified targets for personalized treatments are found to be overexpressed on tumor-associated but not on normal vasculature, like the prostate-specific membrane antigen (PSMA). In BCa, it is expressed on 74% of the tumor vasculature [143].

Radiolabeled PSMA ligands are already widely used in PET imaging of prostate cancer patients, but several preclinical and clinical studies deal with the application of PSMA-PET in BCa [144,145].

A recent study [146] aimed to analyze the potential of [^18^F]flucliclovine and [^68^Ga]Ga-PSMA-11 PET/CT to classify invasive lobular breast carcinoma (NCT04750473). Their primary objective was the improvement of metastasis detection compared to the standard-of-care imaging (CT/bone scan, or FDG). The results demonstrated that the confirmed detection rate was significantly higher for [^18^F]flucliclovine compared to standard-of-care imaging [147]. The mean SUVmax of primary and metastatic lesions was significantly higher for [^18^F]flucliclovine compared to [^68^Ga]Ga-PSMA-11. Especially for combined extra-axillary nodes and distant disease, [^18^F]flucliclovine demonstrated significantly improved detection. Ongoing trials include NCT06059469, NCT04573231, NCT04147494, NCT00967577 and NCT04712721.

Since its discovery in prostate cancer cells, PSMA was quickly implemented in the nuclear medicine field. Prostate cancer exhibits a rather low glucose metabolism [148]; therefore, FDG PET/CT is not the optimal choice for diagnosis. PSMA PET is very precise in detecting pelvic lymphnodes and distant metastases [149]. It is also used to predict not only potential response to [^177^Lu]Lu-PSMA treatment [150], but also for taxane-based chemotherapy [151]. Still, there are some limitations when it comes to PSMA imaging. Cancer cells in solid tumors can have a very heterogeneous PSMA expression, which can be problematic especially in lower grade tumors. Additionally, the spatial resolution of PET/CT is moderate, making it more difficult to detect smaller lesions [152]. ^68^Ga-labeled PSMA is easily accessible, as Ge/^68^Ga generators are widely available.

### 4.3. Immune Cells

Several immune cells are metabolically reprogrammed in the presence of cancers. TAMs can be targeted via several mannose and scavenger receptors, which are overexpressed in some cancers. Macrophage targeting is especially advantageous because it can prevent the development of chemoresistance [13].

The EITHICS phase II study aims to analyze the correlation of inflammation via M1/M2 macrophage polarization in TNBC patients (NCT04320030). In the first step, the patients undergo MRI + FDG PET. After 30 days, macrophage targeting [^18^F]DPA-714 PET/CT is performed followed by subsequent resection of the tumor. The tissue samples are then analyzed for M1/M2 and the 18 kDa translocator protein TSPO expression via IHC. The results of the study revealed that all TNBC patients showed uptake of [^18^F]DPA-714. They conclude that TSPO targeting can be utilized as a specific inflammation marker for TNBC [38].

## 5. Discussion

Molecular markers play a crucial role in BCa imaging, providing valuable information about the biological characteristics of tumors. These specific molecules, proteins, or genetic alterations are associated with certain subtypes of BCa and can predict the behavior of the disease. Some radiotracers like [^18^F]FDG or [^18^F]FES are currently used in clinical practice for the detection and staging of BCa. While [^18^F]FDG is not BCa-specific, it is commonly employed for cancer imaging due to its ability to detect increased glucose metabolism in various cancer cells. On the other hand, [^18^F]FES mimics the structure of estrogen, providing a specific approach to detecting cancers that overexpress the ER. The utilization of [^18^F]FES offers a reliable and precise diagnostic tool for identifying BCa cases characterized by ER overexpression, enhancing the accuracy and efficiency of cancer diagnosis and treatment planning.

As BCa is highly diverse, the importance of tailored and personalized treatments is becoming more evident. While various conventional and experimental therapies have been implemented to address this need, the improvement in individual patient survival, particularly in certain subtypes like TNBC, has been only modest. Novel radiotracers that target specific molecular markers associated with BCa cells hold great promise for improving the accuracy and sensitivity of early cancer detection. [^18^F]FES targeting the ER, [^64^Cu]- or [^89^Zr]Trastuzumab targeting HER2, or [^18^F]FTT targeting PARP enable personalized medicine approaches by tailoring treatment strategies to the individual characteristics of the tumor.

Immuno-PET is a cutting-edge imaging technique combining PET with immune-targeting agents. By using radiolabeled antibodies like [^89^Zr]Trastuzumab, targeting HER2, [^89^Zr]Zr-TLX250, targeting hypoxia or ICIs like [^89^Zr]Zr-atezolizumab, targeting PD-L1 or immuno-PET can provide valuable insights into the immune response within the tumor as well as the TME and help optimize immunotherapy strategies. Various trastuzumab radiotracers underline the significant advancement in nuclear molecular imaging targeting HER2-positive tumors. In general, radiotracers operate at pico- to nanomolar concentrations of targeting molecules, enabling accurate imaging while avoiding the considerable systemic toxicity commonly linked to traditional doses with trastuzumab. Radiolabeled trastuzumab or pertuzumab derivatives can be used in combination with other imaging modalities or therapeutic agents to enhance the accuracy of diagnosis and treatment. This multimodal approach can provide comprehensive information about the tumor and guide treatment decisions.

Overall, the future of radiotracers in combination with chemotherapy or immunotherapy is poised to revolutionize cancer treatment by offering more targeted, effective, and personalized therapeutic approaches. Continued research and clinical trials in this field are essential to further explore the potential synergies and benefits of combining radiotracers with conventional and emerging cancer therapies.

GRPR-targeting radiopeptides seem to be promising, due to the high-density expression in several human cancers and the relatively low physiological expression in healthy tissues. Radiolabeled GRPR antagonists have shown their effectiveness as PET/SPECT/CT tracers in various preclinical and clinical investigations. They have highlighted the benefits of employing a targeted imaging strategy over non-specific tracers like [^18^F] FDG for BCa diagnosis, staging, and restaging [108]. However, despite not directly targeting the ER, PR or HER2 receptor, GRPR radiotracers appear to be a well-suited candidate for BCa theranostics. But the expression of GRPR is closely linked to estrogen expression, suggesting that GRPR radiotracers hold greater promise for the luminal subtypes characterized by ER-positive expression [153].

Radiotracers targeting HR-positive BCa have advanced to clinical phase IV, positioning them as the most promising theranostic agents in this domain. However, despite the continued enhancement of these well-established targets for therapy and imaging, HR-negative BCa continues to present a challenge in radiotracer imaging and treatment. To date, the majority of radiotracers targeting HR-negative BCa are still in clinical phase I or in the beginning of phase II. Overcoming various significant challenges is essential for these radiotracers to advance successfully to clinical phase II trials, particularly concerning radiotracer production, stability, and targeting specificity. A major challenge, spanning multiple medical disciplines, is patient recruitment. The recruitment of suitable patients for clinical trials can be complex, particularly for radiotracers targeting specific biomarkers or patient populations. Ensuring sufficient patient recruitment is crucial for the successful completion of clinical phase II trials.

In addition, the TME plays a crucial role in BCa diagnosis and treatment by influencing tumor growth, progression, response to therapy, and patient outcomes. Tracers like [^68^Ga]PSMA targeting the PSMA expressing endothelial cells or [^68^Ga]FAPI-46 targeting FAP on CAFs allow for a distinctive targeting strategy in BCa imaging, underscoring their exceptional and specialized significance in this domain. A better understanding of the interactions within the TME can lead to the development of more effective and personalized treatment strategies for BCa patients.

The clinical significance and potential impact of these radiotracers lie in their ability to enhance precision oncology by enabling non-invasive, real-time assessment of tumor biology. By targeting specific molecular features, such as receptor status, hypoxic microenvironments, or immune checkpoint expression, these agents might allow for better stratification of patients, improved prediction of therapy response, and more accurate monitoring of disease progression or recurrence as exemplified for a large variety of other malignancies [154]. Importantly, radiotracers not only facilitate diagnosis but increasingly serve as theranostic tools, bridging imaging and therapy within a unified approach. As the field evolves, the integration of molecular imaging into clinical workflows holds the potential to transform standard-of-care in BCa management, particularly in subtypes with limited therapeutic options such as TNBC. Continued clinical validation, technical refinement, and interdisciplinary collaboration will be essential to fully realize the potential of these novel imaging agents in routine clinical practice.

## 6. Conclusions

With advancements in molecular imaging technology and the increasing understanding of cancer biology, researchers are exploring new avenues for the detection of cancer at its earliest stages. The ability to both diagnose and treat cancer at the molecular level offers significant advantages in optimizing patient outcomes and minimizing side effects. The development and refinement of theranostic agents hold great promise for advancing precision medicine in oncology and improving patient care.

In conclusion, this review highlights the significant advancements in the development of novel radiotracers for PET and SPECT nuclear imaging of BCa. This review serves as a necessary contribution to the field, emphasizing the importance of ongoing research and innovation in nuclear molecular imaging for BCa. Some of these new imaging agents summarized and analyzed here offer improved sensitivity and specificity, allowing for non-invasive and whole-body/systemic, accurate detection, characterization, and treatment of breast tumors. However, it is important to acknowledge the limitations of this review, including the focus on specific TNBC molecular markers and the challenges related to patient recruitment for clinical trials. Despite these challenges, the continued exploration and utilization of novel radiotracers will play a crucial role in the fight against BCa. They hold the potential to revolutionize the way we approach BCa management, ultimately leading to better patient outcomes and improved quality of life.

## Figures and Tables

**Table 1:** Current radiotracers used in clinical trials in order of appearance. t.a. = therapeutic approach, n.a. = not applicable; HR = hormone receptor.

Radiotracer	Target	Application	Trial Number	Clinical Phase	Ref.
All tumors					
[^18^F]FDG	Glucose metabolism	PET	NCT04226222 NCT02751710	n.a. n.a.	- [19]
**HR-positive tumors**					
[^18^F]fluoroestradiol	ER	PET	NCT04252859 NCT05541367 NCT05982496 NCT06260033 NCT01273168 NCT04174352 NCT05068726 NCT05486182 NCT06344767 NCT06695039	Phase II n.a. Phase II Phase II Phase I Phase I Phase IV Phase IV n.a. Phase II	- - - - - - - - -
[^18^F]F-4FMFES	ER	PET	NCT04824014	Phase II	[20]
[^68^Ga]Ga-NNS309	ER/PR	PET	NCT06562192	Phase I	-
[^18^F]FFNP	PR	PET	NCT03212170 NCT06086704 NCT06067503	Phase II Phase II Phase II	-
[^89^Zr]Zr-DFO-trastuzumab	HER2	PET	NCT05955833	Phase I	-
[^64^Cu]Cu-DOTA-trastuzumab	HER2	PET	NCT05376878	Phase IV	-
[^68^Ga]Ga-ABY-025	HER2	PET	NCT05619016	Phase II	[21]
[^68^Ga]Ga-NOTA-MVK-ZHER_2:2891_	HER2	PET	NCT05411432	Phase I	[22]
[^99m^Tc]Tc-MIRC208	HER2	SPECT	NCT04591652	n.a.	[23]
[^99m^Tc]Tc-MIRC213	HER2	SPECT	NCT05622240	Phase I	[24]
[^68^Ga]Ga-ABS011	HER2	PET	NCT06369831	Phase II	-
[^177^Lu]Lu-RAD202	HER2	SPECT	NCT06824155	Phase I	-
[^18^F]FDG	Glucose metabolism	PET	NCT03161353	Phase II	[25]
[^68^Ga]Ga-NeoBOMB1	GRPR	PET	EudraCT 2016-002053-38 NCT03724253 NCT03872778	Phase I/IIa	[26,27]
[^64^Cu]Cu-Sarcophagine-Bombesin	GRPR	PET	-	Phase I	[28]
[^99m^Tc]Tc-DB8	GRPR	SPECT	NCT05940298	Phase I	-
[^212^Pb]Pb-DOTAM-GRPR1	GRPR	t.a.	NCT05283330	Phase I	-
[^177^Lu]Lu-NeoB	GRPR	t.a.	NCT05870579 NCT06247995	Phase Ib	-
**HR-negative tumors**					
[^18^F]fluorthanatrace	PARP	PET	NCT03604315 NCT03083288 NCT03846167 NCT05226663	Phase II	[29,30]
[^89^Zr]Zr-TLX250	Hypoxia	PET	NCT04758780	Phase II	[31]
[^177^Lu]Lu-TLX250	Hypoxia	t.a.	NCT05868174	Phase I	-
[^18^F]fluoromisonidazole	Hypoxia	PET	NCT04861077	Phase I	-
[^68^Ga]Ga-Pentixafor	CXCR4	PET	-	n.a.	[32,33,34]
[^89^Zr]Zr-atezolizumab	PD-L1	PET	NCT05742269	n.a.	-
[^99m^Tc]Tc-MY6349	TROP2	SPECT	NCT06104085	n.a.	-
[^68^Ga]Ga-THP-Trop2 VHH	TROP2	PET	NCT06188468	n.a.	-
**[^18^F]F/[^68^Ga]Ga-NOTA-T4**	TROP2	PET	NCT06203574	n.a.	-
[^111^In]In-FPI-1547	IGF-1R	SPECT	NCT03746431	Phase I/II	-
[^225^Ac]Ac-FPI-1434	IGF-1R	t.a.	NCT03746431	Phase I/II	[35,36]
**TME**					
[^68^Ga]Ga-FAPI-46	FAP	PET	NCT06225505 NCT06175390 NCT05574907	Phase II Phase II n.a.	- - -
[^18^F]FDG + [^68^Ga]Ga-FAPI-46	Glucose metabolism + FAP	PET	NCT06335069	Phase II	-
[^18^F]FDG + [^68^Ga]Ga-FAPI-46	Glucose metabolism + FAP	PET	-	-	[37]
[^18^F]FAPI-RGD	FAP + αvβ3-integrin	PET	NCT05976620	n.a.	-
[^68^Ga]Ga-PSMA	PSMA	PET	NCT06059469	Phase II	-
[^18^F]DCFPyL	PSMA	PET	NCT04573231	Phase II	-
[^68^Ga]Ga-PSMA-11 + [^68^Ga]Ga-FAPI-46	PSMA + FAP	PET	NCT04147494	Early phase I	-
Flucliclovine + [^68^Ga]Ga-PSMA-11	ASCT2/LAT1	PET	NCT04750473	Phase I	-
[^68^Ga]Ga-FF58	αvβ3 + αvβ5 Integrins	PET	NCT04712721	Early phase 1	-
[^18^F]DPA714	TSPO	PET	NCT04320030	Phase II	[38]
